# Editorial: Green chemistry biocatalysis

**DOI:** 10.3389/fbioe.2023.1158275

**Published:** 2023-02-20

**Authors:** Gary T. M. Henehan, Barry J. Ryan, Bekir Engin Eser, Ning Li, Zheng Guo, Gemma K. Kinsella

**Affiliations:** ^1^ School of Food Science and Environmental Health, Faculty of Sciences and Health, Technological University Dublin, Dublin, Ireland; ^2^ Department of Engineering, Aarhus University, Aarhus, Denmark; ^3^ School of Food Science and Engineering, South China University of Technology, Guangzhou, China; ^4^ Department of Biological and Chemical Engineering, Faculty of Technical Science, Aarhus University, Aarhus, Denmark

**Keywords:** biocatalysis, cascade reactions, enzyme engineering, green chemistry, hydrogen storage, 3’-phosphoadenosine 5’-phosphosulfate, Penicillin G acylase, enzymatic halogenation

## Green chemistry biocatalysis

There has been an explosion of interest in Green Chemistry Biocatalysis in recent years due to advances in gene manipulation technology, which allow the rapid production of new enzyme variants, as well as advances in the stabilisation of enzymes for biocatalytic processes by immobilisation or mutagenesis. The green agenda which aims to provide industrial processes with a low impact on the environment has added impetus to this area of research.

The potential advantages of enzymes in catalytic terms are their specificity and their ability to work at ambient temperatures in aqueous solutions. However, the use of enzymes in organic chemistry has certain drawbacks: poor stability at high temperatures and poor stability in organic solvents. Moreover, the requirement to operate in an aqueous solution was problematic when high concentrations of poorly soluble reactants were used. A great deal of ingenuity has gone into overcoming these drawbacks (Hanefeld et al., 2022; Miller et al., 2022). The pioneering work of (Klibanov, 2001) showed that enzymes could function in organic media. This, and related work, led to a gradual expansion of the use of enzymes in organic synthesis and today they are increasingly employed to carry out industrial transformations (Bell et al., 2021; Hanefeld et al., 2022; Miller et al., 2022). In recent years, a variety of databases dedicated to biocatalysis have been compiled and software is available to guide retrosynthetic construction of target molecules (Bell et al., 2021).

A key enabler in altering the stability and functionality of enzymes, to allow them to operate on novel substrates, for example, was site directed mutagenesis combined with molecular modelling tools. Mutagenesis can be used to make enzymes more stable to solvents or to high temperatures as well as altering active site specificity. Enzyme stability may be further improved by immobilisation on supports where, due to multiple points of attachment, a protein chain is more resistant to denaturation (Priyanka et al., 2019; Hanefeld et al., 2022). More recently, an awareness that enzymes can catalyse reactions in ionic liquids and deep eutectic solvents has led to further expansion of the range of applications of enzymes in organic synthesis (Yu et al., 2022; Arnodo et al., 2023).

A further development of biocatalysis lies on the application of enzyme cascade reactions. These reactions involve more than one enzyme carrying out reactions in sequence. The challenges of such systems lie in the compatibility of substrates with the enzymes used and the recovery of product from complex mixtures. Despite the challenges, considerable progress in multi-step enzymatic synthesis have been made (Bell et al., 2021; Hanefeld et al., 2022).

The papers listed in this Research Topic provide interesting examples of the use of enzymes in synthesis (see below).

### Biocatalytic process for hydrogen storage

In this report, authors Cha et al. describe an enzyme system for trapping Hydrogen gas as formic acid. The development of renewable energy technologies to replace fossil fuels is essential for the sustainable growth of the economy and society. While Hydrogen is an alternative fuel with high gravimetric energy density and net-zero carbon dioxide (CO_2_) production there are current limitations to its transportation and storage as a fuel. Cha et al. demonstrated the conversion of H_2_ and CO_2_ into formate, a non-flammable liquid at ambient temperature and pressure which is more convenient to transport and store than hydrogen gas. This was achieved using an NAD^+^-dependent cascade reaction of an O_2_-tolerant hydrogenase (H_2_ase) and formate dehydrogenases (FDH) in the presence of O_2_ albeit with further optimisation required in future studies.

### A novel cascade reaction for the synthesis of a key coenzyme

Sulfation is an essential biological process for regulating the bioactivity of many compounds. Monterrey et al., explored the development of a novel, efficient and sustainable sulfation process. Sulfotransferases are dependent on the coenzyme 3′-phosphoadenosine 5′-phosphosulfate (PAPS), which is expensive and difficult to obtain. Monterrey et al. developed a modular multienzyme system to allow the *in-situ* synthesis of PAPS and its coupling to a chondroitin sulfation system in an approach that will likely underpin future work in this area.

### Towards the biocatalytic synthesis of antibiotics

Pan et al. have provided an interesting overview of progress towards the biocatalyic synthesis of β-lactam antibiotics by Penicillin G acylase (PGA). This enzyme is the second most widely used in the world for commercial synthesis. PGA was first isolated in the 1950s and has been widely used for the hydrolysis of Penicillin G to produce 6-Aminopenicillanic acid, a precursor of other penicillins. This hydrolysis reaction catalysed by PGA can be reversed by use of alternative acyl donors to produce semi-synthetic penicillins. The success of such reactions depends on a balance between hydrolytic and synthetic activities of the enzyme. In this respect, the enzyme still lacks efficiency for large scale synthesis applications. This review examines the strategies used to improve PGA performance in the last 20 years such as the use of bioprospecting for improved PGA variants, solvent engineering, *in situ* product removal, and the use of a one-pot reaction cascade. These advances provide important guidelines for the future use of enzymatic synthesis and possible combinations of strategies for the industrial production of β-lactam antibiotics.

### Novel biocatalytic halogenation of arenes

Li et al. report a nice enzymatic method for the regioselective C-H halogenation of arenes, a valuable synthetic transformation. They demonstrate that a thermostable formate dehydrogenases (FDH), a tryptophan 7-halogenase variant named as 3-LSR, can carry out efficient regioselective monobromination of various indole, azaindole and anthranilamide compounds. Although FDHs require continuous regeneration of their FADH_2_ by a flavin reductase, the authors unexpectedly observed efficient enzymatic bromination without the addition of a reductase partner. Further investigation revealed that 3-LSR was utilizing a flavin reductase from its expression host *E. Coli*. Thus, an initially unintended co-purification of the reductase enables a simpler reaction system for this important biocatalytic transformation.

## Conclusion

The strategies described in this Research Topic provide interesting examples of the use of enzymes in industrially important reactions. The applications of enzymes in industrial biocatalytic synthesis are undergoing a revolution at present and the range of reactions now possible is impressive, rivalling traditional chemical synthesis (Hanefeld et al., 2022). This approach to synthesis of chemical entities will only gain momentum in the coming years and has the potential to transform our lives and the environment.
